# Enhanced matrix inference with Seq2seq models via diagonal sorting

**DOI:** 10.1038/s41598-023-50919-2

**Published:** 2024-01-09

**Authors:** Wei Peng, Yisong Wang, Maonian Wu

**Affiliations:** 1 Department of Computer Science, Guizhou University, Jiaxiu South Road, Guiyang, 550000 Guizhou China; 2 State Key Laboratory of Public Big Data, College of Computer Science and Technology, Guizhou University, Jiaxiu South Road, Guiyang, 550000 Guizhou China; 3 The Information Engineering College, Huzhou University, 2nd Ring Road East, Huzhou, 313000 Zhejiang China; 4 Zhejiang Province Key Laboratory of Smart Management and Application of Modern Agricultural Resources, Huzhou University, 2nd Ring Road East, Huzhou, 313000 Zhejiang China

**Keywords:** Computer science, Computational science

## Abstract

The effectiveness of sequence-to-sequence (seq2seq) models in natural language processing has been well-established over time, and recent studies have extended their utility by treating mathematical computing tasks as instances of machine translation and achieving remarkable results. However, our exploratory experiments have revealed that the seq2seq model, when employing a generic sorting strategy, is incapable of inferring on matrices of unseen rank, resulting in suboptimal performance. This paper aims to address this limitation by focusing on the matrix-to-sequence process and proposing a novel diagonal-based sorting. The method constructs a stable ordering structure of elements for the shared leading principal submatrix sections in matrices with varying ranks. We conduct experiments involving maximal independent sets and Sudoku laws, comparing seq2seq models utilizing different sorting methods. Our findings demonstrate the advantages of the proposed diagonal-based sorting in inference, particularly when dealing with matrices of unseen ranks. By introducing and advocating for this method, we enhance the suitability of seq2seq models for investigating the laws of matrix inclusion and exploring their potential in solving matrix-related tasks.

## Introduction

Deep learning has achieved significant success in natural language processing (NLP)^1^. In particular, many tasks, such as machine translation, text summarization, and dialogue systems can be represented as sequence-to-sequence (seq2seq) form^2^, which takes a sequence as input (e.g., the sequence of sentences to be translated in machine translation) and produces another sequence as output (i.e., the sequence of translated sentences), thus learning the laws between the sequences by using the seq2seq model, and to accomplish the particular task. Representatively, to efficiently utilize contextual information, Vaswani et al.^3^ proposed the Transformer by introducing the attention mechanism into seq2seq models and achieved state-of-the-art results in the machine translation.

Since the success of seq2seq models in NLP, several studies have extended their utility by treating complex computational tasks as instances of machine translation. Examples include symbolic integration, solving mathematical word and geometry problems, and other tasks requiring sophisticated inference^4–6^. These works have achieved impressive results by representing inputs like mathematical equations as sequences and training seq2seq models to learn the transformation laws mapping between input and output sequences. Matrices are a fundamental representation tool for organizing numbers, symbols, or expressions in rows and columns^7,8^. They play a crucial role in various fields, including deep learning, where generalized matrices (or tensors) serve as the fundamental computational units for many popular methods^9^. Matrix operations are also essential in application fields such as programming problems, image processing, and information encryption^10–12^. From a structural representation perspective, matrices can be transformed into sequences by arranging their elements, making them a suitable input for seq2seq models. However, the current matrix-to-sequence process heavily relies on generic row-based sorting (RS) and column-based sorting (CS). Consequently, there is a need to explore suitable matrix-to-sequence methods that can unlock the computational potential of the seq2seq model and effectively address tasks represented by matrices.

Deep learning has demonstrated a remarkable ability to generalize by effectively partitioning datasets during training, which enables models to make inferences on previously unseen data. However, the presence of matrix rank poses a significant challenge to the generalization performance of the models. More specifically, when confronted with matrices of unseen rank, seq2seq models with RS/CS struggle to accurately predict sequences. Taking inspiration from Cantor’s diagonal argument^13^, this study proposes diagonal-based sorting (DS) as a method to implement the matrix-to-sequence process. By constructing sequential representations of matrices using DS, the seq2seq model learns the certain laws of the matrix dataset in a generalized manner, facilitating accurate sequence generation even for matrices with unseen rank. To demonstrate the necessity of our method, we conduct exploratory experiments. We then compare the effects of different sorting methods on the seq2seq model through experiments involving maximum independent sets and Sudoku, providing experimental results that validate the effectiveness of our proposed method. The contributions of this study are summarized as follows.

- We proposed a novel DS method to convert matrices into sequences for input into seq2seq models. DS constructs stable ordering of elements for shared leading principal submatrix sections across matrices of varying ranks. This allows seq2seq models to make accurate inferences even on matrices of unseen ranks.
- We validate DS and highlight the significance of mapping invariance on complex matrix inference tasks involving maximum independent sets and Sudoku.
- We provide open access to all the datasets, models, and corresponding code utilized in this research.

The remainder of the paper is organized as follows. “Method” presents our method, and “Experiments” describes the experiments and the analysis of the results. “Related works” discusses related works. Finally, conclusions and future work are given in “Conclusions”.

## Method

**Figure 1:**
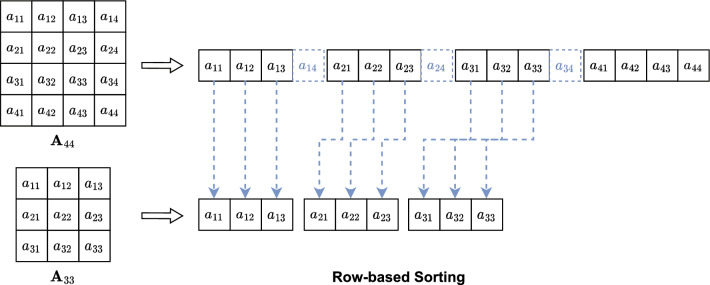
Schematic illustration of matrices converted to a sequences by row-based sorting.

### Limitations of row (column)-based sorting in Seq2seq

RS and CS are widely employed in the fields of computation, storage, communication, and others to efficiently convert matrices into sequences. To accommodate dimensional restrictions, it is customary to use the notation [[a,b],[c,d]] instead of $$\begin{pmatrix} a &{} b\\ c &{} d \end{pmatrix}$$when representing 2nd-order matrices. In this notation, [a,b] and [c,d] represent the rows of the matrix. Moreover, these methods are commonly utilized to stretch tensors, which represent data, into vectors for input into deep learning models. This is done to facilitate batch processing and computation.

Figure 1 presents a schematic diagram illustrating the transformation of matrices of varying orders through RS to generate a sequence. We represent the elements of the matrices using small squares. With RS, the matrices of rank 4 and 3 (left side of the figure) are transformed into two sequences (right side of the figure), respectively. The first sequence consists of every fourth consecutive square, representing the rows of the matrix. Similarly, the second sequence is composed of every third consecutive square.

Such sorting methods may appear reasonable, but they are unsuitable for deep learning models, particularly for seq2seq models designed to fit data with matrix properties. This inadequacy arises because matrices of different ranks cannot maintain a consistent order within the corresponding sequences. Figure 1 serves as an illustration of this issue, showcasing two matrices with elements $a_{21}$ in the first column of the second row. Despite occupying identical positions within their respective matrices, these elements are shifted within the sequences.

When utilizing RS, the seq2seq model encounters difficulties when inferring on matrices with varying ranks. For instance, during training, the model effectively predicts the transpose matrix of a specific 10th-order matrix. However, during the inference process, the test data is a 5th-order leading principal submatrix extracted from the matrix, where leading principal submatrix denotes a submatrix composed of the initial *n* rows and columns of a matrix. Without a clear understanding of the concept of matrix rank, the model struggles to comprehend the need for shifting certain elements to the front in the output sequence, as previously discussed. In “Exploratory experiments”, we will provide additional validation to support this idea through exploratory experiments.

### Diagonal-based sorting

To ensure optimal performance of the trained seq2seq model in matrix inference, it is essential to employ appropriate sorting methods that facilitate the conversion of matrices with varying orders into sequences with consistent element ordering. In order to outline this objective, Definition 1 is introduced to formalize the property for a matrix-to-sequence method.

#### Definition 1

(*Mapping invariance*) Let *f* be a matrix-to-sequence method that maps the elements of a matrix to the elements of the sequential representation of the matrix. *f* is mapping invariance if for matrix ${\textbf{A}}=(a_{ij})_{k_{1},k_{1}}$ such that

$$\begin{aligned} S_{{\textbf{A}}}(l)=S_{{\textbf{B}}}(l), 1 \le l \le k_{2}\times k_{2}, \end{aligned}$$

where ${\textbf{B}}=(b_{ij})_{k_{2},k_{2}}$ is a leading principal submatrix of ${\textbf{A}}$. $S_{{\textbf{A}}}$ and $S_{{\textbf{B}}}$ are the sequential representations of **A** and **B**, respectively, by *f*.

Definition 1 employs the concept of a leading principal submatrix to elucidate mapping invariance. In particular, Example 1 is utilized to demonstrate that RS fails to exhibit mapping invariance.

#### *Example 1*

Let $${\textbf{A}}= \begin{pmatrix} 2 &{} 3 &{} 4\\ 0 &{} 5 &{} 5\\ 7 &{} 0 &{} 3 \end{pmatrix}$$ be a matrix and $${\textbf{B}} =\begin{pmatrix} 2 &{} 3\\ 0 &{} 5 \end{pmatrix}$$ is a 2nd-order leading principal submatrix of ${\textbf{A}}$. The sequential representation $S_{{\textbf{A}}}=\mathtt {[2,3,4,0,5,5,7,0,3]}$ and $S_{{\textbf{B}}}=\mathtt {[2,3,0,5]}$ obtained by RS. It can be noticed that the third element of $S_{{\textbf{A}}}$ and $S_{{\textbf{B}}}$ appears different, although matrix ${\textbf{A}}$ and matrix ${\textbf{B}}$ have the same elements within the second row and the second column. Therefore, RS does not satisfy the mapping invariance and CS is in a similar situation.

**Figure 2:**
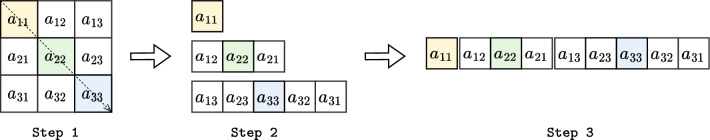
Overview of diagonal-based sorting.

The strategy of sorting matrix elements is introduced in Cantor’s diagonal argument, which demonstrates that the existence of uncountable sets can be proven by arranging the diagonal elements of a matrix^13^. Specifically, Cantor considered representing real numbers in a table where each row lists the digits of a decimal expansion. He then constructed a new number by taking diagonal elements from this table, choosing a different digit in each position. This newly constructed “diagonal” number differs from every number in the table, proving that trying to list all real numbers leads to a contradiction.

Our work draws inspiration from this idea of utilizing the diagonals of a matrix. We proposes a straightforward and efficient method for converting matrices to sequences, known as DS, to enhance the seq2seq model. By utilizing DS to organize matrices into sequences prior to training the seq2seq model, one can ensure a certain performance even when inferring about matrices with previously unseen order.

We begin by presenting an overview of DS in Fig. 2. This process consists of three steps. In Step 1, DS divides the matrix into multiple sub-blocks using the main diagonal elements as clues. In Step 2, the elements within each sub-block are sorted in the same direction to obtain corresponding sequences. Finally, in Step 3, all the sub-block sequences are concatenated to create the sequential representation of the matrix. Formally, DS constructs the sequence $S^{i}_{{\textbf{M}}}$ centered on each diagonal element $m_{ii}$ for an *n*th-order square matrix ${\textbf{M}}=(m_{ij})_{n,n}$. The sequential representation $S_{{\textbf{M}}}$ is obtained by concatenating all $S^{i}_{{\textbf{M}}}$ after iterating through all diagonal elements, as shown in Eq. (1).

1 $$\begin{aligned} \begin{aligned} S^{1}_{{\textbf{M}}}&=[\mathtt {m_{1,1}}]\\ S^{2}_{{\textbf{M}}}&=[\mathtt {m_{1,2},m_{2,2},m_{2,1}}]\\ S^{i}_{{\textbf{M}}}&=[\mathtt {m_{1,i},m_{2,i},\ldots ,m_{i-1,i},m_{i,i},m_{i,i-1},\ldots ,m_{i,2},m_{i,1}}]\\ S_{{\textbf{M}}}&=S^{1}_{{\textbf{M}}} \oplus S^{2}_{{\textbf{M}}} \oplus \cdots \oplus S^{i}_{{\textbf{M}}} \oplus \cdots \oplus S^{n}_{{\textbf{M}}} \end{aligned} \end{aligned}$$

where $\oplus$ is the connection operation.

The bi-directionality of the encoding process of DS is evident. This process can be used to sort a given square matrix into sequences, and conversely, by inverting the process, sequences of length $n^2$ can be encoded to form square matrices of *n*th order.

The utilization of DS is significant as it guarantees mapping invariance, ensuring that sequences corresponding to square matrices of different ranks maintain a consistent order of elements. To illustrate, let’s consider a matrix, ${\textbf{A}}=(a_{ij})_{k_{1},k_{1}}$, and its leading principal submatrix, ${\textbf{B}}=(b_{ij})_{k_{2},k_{2}}$. The elements of the common parts of the two matrices have the same ordering in the sequence obtained by DS. Specifically, for each *i* from 1 to $k_{2}$, the sequence $S^{i}=\mathtt {[a_{1,i},a_{2,i},\ldots ,a_{i-1,i},a_{i,i},a_{i,i-1},\ldots ,a_{i,2},a_{i,1}]}$ maintains this consistent order. Example 2 is used for supplementary illustration.

#### *Example 2*

Same as example 1, let $${\textbf{A}}=\begin{pmatrix} 2 &{} 3 &{} 4\\ 0 &{} 5 &{} 5\\ 7 &{} 0 &{} 3 \end{pmatrix}$$ be a matrix and $${\textbf{B}}=\begin{pmatrix} 2 &{} 3\\ 0 &{} 5 \end{pmatrix}$$ is a 2nd-order leading principal submatrix of ${\textbf{A}}$. In DS process, there is $S_{{\textbf{A}}}^{1}=S_{{\textbf{B}}}^{1}=[\texttt{2}]$, $S_{{\textbf{A}}}^{2}=S_{{\textbf{B}}}^{2}=[\texttt{3,5,0}]$; therefore, the same part of ${\textbf{A}}$ and ${\textbf{B}}$ can correspond to invariant indices in the sequential representations.

### Exploratory experiments

This section presents exploratory experiments aimed at examining the impact of DS in the inference process of the seq2seq model. We employ the Transformer model^3^ to learn the law of transposition of matrices. These experiments are relatively fundamental, as they solely involve changes in the position of elements according to the mapping rules. Nonetheless, they provide an appropriate context for analyzing the impact of DS. For the sake of conciseness, we use the terms *transpose* in italics to denote the exploratory experiments.

The matrices ${\textbf{A}}=(a_{ij})_{m,m}$ and ${\textbf{B}}=(b_{ij})_{m,m}$ serve as the inputs and outputs, respectively, of *transpose*, and naturally, ${\textbf{A}}$ and ${\textbf{B}}$ are mutually transposed, meaning that $a_{ij}=b_{ji}$. To demonstrate the benefits of DS in the inference process of the seq2seq model, we randomly generated 1000 pairs of such transposed matrices $({\textbf{A}},{\textbf{B}})$, and incorporated varying matrix ranks in both the training and inference stages. Initially, we trained the model using matrices of rank 20/30, which were divided into a training set and a test set in an 8:2 ratio. Subsequently, the inference process was carried out on 1000 matrices of different ranks.

**Table 1:** The results of the transposition experiments.

Ranks	20 (train) (%)	19 (%)	18 (%)	17 (%)	15 (%)	12 (%)	10 (%)
$$\text {ACC}_\text {single}$$							
RS	100	10.3	10.2	10.9	10.6	11.1	11.9
CS	100	10.3	10.2	10.9	10.5	11.6	11.3
**DS**	100	100	100	100	99.9	99.9	99.9
**c-DS**	100	100	100	100	99.9	99.9	99.9

Ranks	30 (train) (%)	29 (%)	28 (%)	27 (%)	25 (%)	22 (%)	20 (%)
$$\text {ACC}_\text {single}$$							
RS	99.9	10.1	10.1	10.2	10.1	10.3	10.5
CS	100	10.1	10.1	10.1	10.1	10.3	10.8
**DS**	99.9	99.9	99.9	99.9	99.9	99.9	99.9
**c-DS**	100	99.9	99.9	99.9	99.9	99.9	99.9

Ranks	20 (train) (%)	19 (%)	18 (%)	17 (%)	15 (%)	12 (%)	10 (%)
$$\text {ACC}_\text {total}$$							
RS	100	0.0	0.0	0.0	0.0	0.0	0.0
CS	100	0.0	0.0	0.0	0.0	0.0	0.0
**DS**	100	100	100	100	99.8	98.7	98.5
**c-DS**	100	100	100	100	99.8	98.7	98.5

Ranks	30 (train) (%)	29 (%)	28 (%)	27 (%)	25 (%)	22 (%)	20 (%)
$$\text {ACC}_\text {total}$$							
RS	99.9	0.0	0.0	0.0	0.0	0.0	0.0
CS	100	0.0	0.0	0.0	0.0	0.0	0.0
**DS**	99.9	99.8	99.7	99.9	99.8	99.5	99.5
**c-DS**	100	99.9	99.9	99.9	99.9	99.9	99.9

RS, CS, DS, and c-DS represent the sorting methods utilized. $\text {ACC}_\text {single}$ indicates the percentage of accurately predicted individual matrix elements, while $\text {ACC}_\text {total}$ signifies the percentage of complete matrices that were correctly predicted.

The outcomes of the *transpose* are presented in Table 1. In Table 1, we present a supplementary sorting method called counter-DS (c-DS) to demonstrate the extension of Definition 1. The c-DS involves sorting the elements of the sub-blocks in the reverse direction of DS during the sorting process. For an *n*th-order square matrix ${\textbf{M}}=(m_{ij})_{n,n}$, c-DS constructs the sequence $S_{{\textbf{M}}}$ according to Eq. (2). Notably, c-DS still adheres to the mapping invariance outlined in Definition 1. To enhance clarity, bold formatting is employed in the tables to indicate the sorting methods that adhere to mapping invariance, specifically DS and c-DS.

2 $$\begin{aligned} \begin{aligned} S^{1}_{{\textbf{M}}}&=[\mathtt {m_{1,1}}]\\ S^{2}_{{\textbf{M}}}&=[\mathtt {m_{2,1},m_{2,2},m_{1,2}}]\\ S^{i}_{{\textbf{M}}}&=[\mathtt {m_{i,1},m_{i,2},\ldots ,m_{i,i-1},m_{i,i},m_{i-1,i},\ldots ,m_{2,i},m_{1,i}}]\\ S_{{\textbf{M}}}&=S^{1}_{{\textbf{M}}} \oplus S^{2}_{{\textbf{M}}} \oplus \cdots \oplus S^{i}_{{\textbf{M}}} \oplus \cdots \oplus S^{n}_{{\textbf{M}}} \end{aligned} \end{aligned}$$

The accuracy metrics used to evaluate the model are $\text {ACC}_{\text {single}}$ and $\text {ACC}_{\text {total}}$, as presented in Table 1. $\text {ACC}_{\text {single}}$ represents the accuracy of correctly predicting a single element of the input matrix, while $\text {ACC}_{\text {total}}$ measures the success of predicting a complete matrix, considering it correct only when all elements within the matrix have been accurately predicted.

Through a horizontal analysis of the results depicted in Table 1, we commence the model training phase using matrices of 20/30 rank. Subsequently, we progressively reduce the rank of the matrices to assess the model’s inference performance. Evaluation based on both the $\text {ACC}_\text {single}$ and $\text {ACC}_\text {total}$ metrics reveals that the seq2seq model, utilizing RS/CS, successfully predicts only a fraction of the matrix elements, failing to accurately predict the entire matrix. However, when DS/c-DS is employed as a sorting method instead, the seq2seq model achieves remarkable accuracy on the test matrices.

**Figure 3:**
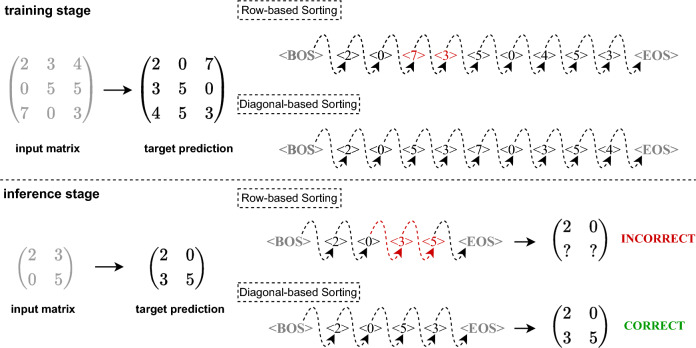
Schematic illustration for analysis of transposition experiment results.

We analyze the relationship between the *transpose* results and the mapping invariance described in Definition 1 using Fig. 3. During the training phase, the model captures features from input matrices of known ranks and successfully predicts the corresponding target matrices. However, in the inference phase, the accuracy of the model’s autoregressive predictions is heavily influenced by the sequence order of tokens. To illustrate, consider the example depicted in Fig. 3. While the model accurately fits a certain pair of transposed matrices at the 3rd order during training, it struggles to predict the 2nd order leading principal submatrices of the same matrix during inference. This is due to the disruption in the sequence order caused by RS, as elements are removed when reducing the matrix rank, violating the mapping invariance. In contrast, using DS maintains the consistent order of elements in the sequences, ensuring that corresponding elements in the target matrices align properly. Considering the findings in Table 1, it is evident that RS/CS primarily predicts the elements in the first row/column of the target matrix, while DS/c-DS accurately predicts the entire matrix.

## Experiments

### Experimental environment

To further assess the impact of DS on the seq2seq model, we performed experiments using more complex datasets in addition to those previously discussed in “Exploratory experiments”,. Specifically, we explored sequence prediction tasks such as Maximum Independent Set and Sudoku. The experimental environment was set up with an NVIDIA Tesla V100-SXM2 GPU with 32 GB of memory and the Debian 8.3.0 operating system. For implementation, we utilized Python 3.8 and PyTorch 1.10, along with NetworkX 2.8 for constructing graph-related datasets. All codes used for reproduction can be accessed in the https://github.com/Peng-weil/ds-for-seq2seq. All the experimental results are averaged over 5 independent runs. It is important to note that the matrices requiring diagonal sorting in our experiments were predominantly square matrices. In cases where non-square matrices were encountered, we ensured a square matrix shape by populating it with special characters.

The experiments utilized the standard Transformer model as the seq2seq model, incorporating the following hyperparameters^3^:

- *8* attention heads,
- *6* encoder and decoder layers,
- *256* embedding dimension,
- Adam optimizer with ${10}^{{-4}}$ learning rate.

### Maximum independent set

#### Dataset

**Figure 4:**
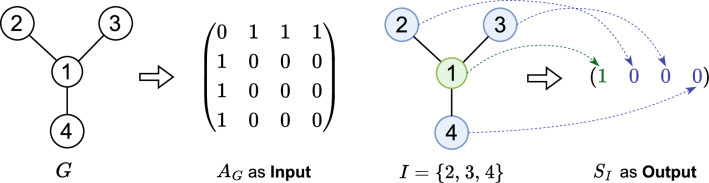
Illustration of the dataset for the maximum independent set experiment, where the adjacency matrix $A_{G}$ of one of a graph *G* is the input of the model and $S_{I}$ is the output of the model.

The concept of the maximum independent set holds significant importance in the field of graph theory. It refers to a collection of disconnected nodes in a graph, and adding any new node from the graph to this set creates a connection between some two vertices in the set. Formally, Let $G=(V,E)$ be an undirected graph, where *V* represents the set of vertices and *E* represents the set of edges. An independent set, denoted as *I*, is a subset of *V* in which no two vertices are adjacent (i.e., there is no edge connecting any pair of vertices in *I*). The maximum independent set, denoted as $I_{max}$, is the largest possible independent set within the given graph G, it is an independent set with the greatest number of vertices. The size of $I_{max}$ is denoted as $|I_{max}|$, which represents the cardinality or the number of vertices in the maximum independent set. The problem of finding the maximum independent set is known as the “Maximum Independent Set problem”, which is a well-known NP-hard problem in computer science. It provides insights into the structure and connectivity of graphs and plays a crucial role in algorithm design and analysis.

The dataset $D_{MIS}=(A_{G},S_{I})$ is created to characterize the transformation of the maximum independent set, as depicted in Fig. 4. Here, $A_{G}$ denotes the adjacency matrix of a graph *G*, and $S_{I}$ represents the maximum independent set of *G* in the form of a sequence. To ensure semantic consistency with the elements in $A_{G}$, when the value in $S_{I}$ is 0, it signifies that the corresponding node at that position index is independent and not connected. Conversely, when the value is 1, it indicates the opposite. The NetworkX library is utilized for computing the maximum independent set of graph *G* to guarantee dataset accuracy. Furthermore, we assign the unique label to the maximum independent set that has the earliest sequential order according to the node numbers.

#### Results

**Table 2:** The results of the maximum independent set experiments with 16-node graph training on 200 K data and 25-node graph training on 400 K data.

Nodes	16 (train) (%)	15 (%)	14 (%)	13 (%)	12 (%)
ACC_single_					
RS	99.9	67.93	65.08	63.53	62.45
CS	99.98	67.83	66.14	64.9	64.25
** DS**	99.99	99.78	99.81	98.51	95.84
** c-DS**	99.99	99.8	99.73	99.81	99.08

Nodes	25 (train) (%)	24 (%)	23 (%)	22 (%)	21 (%)
RS	99.98	67.91	63.24	62.83	61.14
CS	99.37	62.34	59.18	57.27	58.6
** DS**	99.95	99.56	98.88	97.11	96.98
** c-DS**	99.87	99.34	98.74	98.57	97.69

Nodes	16 (train) (%)	15 (%)	14 (%)	13 (%)	12 (%)
ACC_total_					
RS	99.99	0.11	0.05	0.1	0.15
CS	99.83	0.15	0.13	0.27	0.4
**DS**	99.99	96.86	97.56	85.29	69.82
** c-DS**	99.98	97.14	96.62	97.71	90.14

Nodes	25 (train) (%)	24 (%)	23 (%)	22 (%)	21 (%)
RS	97.6	0.16	0.14	0.06	0.08
CS	98.34	0.07	0.11	0.15	0.1
**DS**	98.96	90.74	77.67	75.24	68.52
**c-DS**	97.19	85.87	74.53	76.77	66.41

The performance of the seq2seq model with various sorting methods in the Maximum Independent Set experiment is presented in Table 2. In line with the experiments mentioned in “Exploratory experiments”, the initial training is conducted on a 16/25-node graph, which is subsequently stepwise reduced in size to assess the inference performance of the models using $\text {ACC}_\text {single}$ and $\text {ACC}_\text {total}$ as evaluation metrics.

The seq2seq models employing four distinct sorting methods demonstrate exceptional accuracy when tested on a graph containing the same number of nodes as the training data graph. However, as the number of nodes decreases, the RS/CS method struggles to accurately predict the label sequence, despite the sequence length being a mere 12–15 tokens. On the other hand, the DS/c-DS method maintains a certain level of accuracy, albeit with a noticeable decline as the number of nodes decreases. It is worth noting that in both the 16 node and 25 node experiments, the model’s accuracy decreases as the difference between the seen matrix rank and the unseen matrix rank increases. This indicates that as the gap widens between test data and training data, the model finds it increasingly difficult to leverage patterns learned during training. However, methods that do not guarantee mapping invariance exacerbate the problem due to elements shifting to other positions. By contrast, DS maintains consistency in element ordering, preserving training generalizability as much as possible. Table 2 showcases a discernible trend indicating that the performance of the seq2seq model is significantly influenced by encoding the input data into an appropriate sequence prior to training.

### Sudoku

#### Dataset

Sudoku is a logic-based number puzzle that has gained worldwide popularity. The game is played on a 9 $\times$ 9 grid, composed of nine 3 $\times$ 3 boxes. The objective of the game is to fill the grid with the numbers 1–9, ensuring that each number appears only once. The puzzle follows three primary rules: each row and column must contain the numbers 1–9, and each 3 $\times$ 3 box must also include these numbers.

Numerous studies have utilized neural networks to solve Sudoku puzzles^14,15^. The input for a Sudoku dataset typically consists of a 9 $\times$ 9 matrix with numbers ranging from 0 to 9. In this representation, 0 signifies an empty cell that requires filling to conform to the Sudoku rules, while numbers 1–9 denote cells already filled with respective values. The output is a 9 $\times$ 9 Sudoku solution wherein each cell contains a number from 1 to 9. The Sudoku dataset can be effectively utilized for training a seq2seq model. In this section, we utilize a dataset of 100 K Sudoku puzzles to evaluate the effectiveness of the seq2seq model in learning Sudoku patterns across various sorting methods.

#### Results

**Table 3:** The results of the Sudoku experiments with 100 K puzzles.

	Training set (%)	Test set (%)
$$\text {ACC}_\text {single}$$		
RS	99.93	96.49
CS	99.63	96.21
**DS**	99.99	99.67
**c-DS**	99.99	99.54
$$\text {ACC}_\text {total}$$		
RS	94.8	10.30
CS	95.8	16.32
**DS**	99.14	62.86
**c-DS**	99.0	57.75

The Sudoku dataset of size 100 K is divided into training and testing sets in an 8:2 ratio. The seq2seq model is then trained using four sorting methods, and the results are presented in Table 3. Both seq2seq models, under RS/CS and DS/c-DS, demonstrate high accuracy in predicting individual elements on the test set. This indicates that both sorting strategies effectively accommodate the features of Sudoku data and utilize them for predictions. It is important to note that the presence of numbers from the puzzle in some Sudoku puzzles leads to higher accuracy in predicting individual elements. However, when it comes to predicting an entire puzzle correctly, the seq2seq model with DS/c-DS exhibits higher accuracy compared to RS/CS. This could be because DS compacts the first 3 $\times$ 3 section of the Sudoku, thereby facilitating the learning of attentional mechanisms.

## Related works

A highly relevant approach is to encode numbers as symbolic sequences and use text-based architectures like Transformer for training and inference, which can be seen as a neural-symbolic method. Saxton et al.^16^ brought this problem into view earlier by proposing a dataset containing various mathematical problems and conducting large-scale benchmark tests on Transformer models and LSTMs with attention mechanisms, demonstrating these models can achieve moderate performance on some mathematical problems. The study by Lample et al.^4^is particularly relevant to our research as they employ the Transformer model to perform symbolic operations, including differentiation, integration, and solving ordinary differential equations. In order to train the model using mathematical expressions, they use prefix encoding to convert formulas into sequences that can be processed by the model. Lewkowycz et al.^17^trained a large language model called Minerva that is capable of solving middle school to college level math and science problems without relying on external tools, by continuing pretraining it on technical subject data. Their model achieves state-of-the-art results on several quantitative reasoning benchmark datasets. Faber and Wattenhofer^18^ proposed a novel neural architecture called Neural Status Registers tailored for learning comparisons between numbers, which can be combined with other models to solve interesting problems requiring comparisons, such as piecewise-defined functions, image digit comparison, recurrent computation like finding minimum elements, and more. The key difference between the aforementioned works and our work is that we process more abstract data with a specific focus on matrix-type data. More specifically, the input data in our case can be represented in matrix form where each element has certain relationships with other elements in its row and column. Moreover, we design a novel matrix-to-sequence encoding tailored for seq2seq models. This enables the models to achieve reasonable prediction performance even when tested on matrices of unseen ranks.

The permutation invariance in set-to-sequence paradigm provides inspiration for proposing mapping invariance in this study. With the set-to-sequence approach, the input consists of an unordered collection of elements, while the output is an ordered sequence^19^. Unlike the widely used seq2seq method, the set-to-sequence approach needs to address the appropriate representation of the input set, given its unordered nature.

In recent years, the integration of Transformer models with mathematical problems has become a significant focus of research^20–25^. For example, d’Ascoli et al.^26^train a Transformer model to infer functions and recursive relationships for integer or floating-point number sequences. Their evaluation, conducted on a subset of OEIS sequences, demonstrates the model’s superior performance compared to built-in Mathematica functions in recursive prediction. Popov et al.^27^propose a novel deep learning framework that utilizes a variational autoencoder to generate symbolic expressions. Their approach outperforms contemporary symbolic regression benchmarks, especially in noisy conditions. Additionally, Cornelio et al.^28^highlight the derivation of models for natural phenomena from axiomatic knowledge and experimental data through the fusion of logical inference and symbolic regression. Mandlecha et al.^29^proposed a hybrid tokenization technique to encode mathematical and science problems which requires less memory and achieves better accuracy compared to character-level tokenization in several domains. They also introduced an extensive dataset of mathematical and science problems spanning topics like calculus, linear algebra, mechanics, optics, etc. Frieder et al.^30^ assess ChatGPT’s mathematical capabilities using publicly available and handcrafted datasets and compare its performance to other models trained on mathematical corpora, such as Minerva. They also simulate various use cases that mathematicians encounter in their daily professional activities, such as question-answering and theorem searching, to determine if ChatGPT can serve as a useful assistant for professional mathematicians.

## Conclusions

Numerous studies have underscored the potential of seq2seq models in addressing mathematical problems such as calculus, geometry, and mathematical word problems. Despite these achievements, seq2seq models employing generic sorting methods have encountered difficulties in comprehending the specific rules embedded within matrix data. Specifically, these models have struggled to make accurate inferences over matrices with unseen rank, resulting in less optimal outcomes. To overcome these limitations, this research delves into the matrix-to-sequence process of seq2seq models and proposes DS as a solution for attaining sequential representations of matrices. DS guarantees a consistent ordering of elements in the shared leading principal submatrices across matrices of various orders. Such consistency is paramount for precise inference in seq2seq models. This study presents an exploratory experiment that emphasizes the urgency of introducing DS. Additionally, maximum independent set and sudoku experiments are conducted to illustrate the advantages of DS compared to generic sorting methods. By harnessing DS, seq2seq models enhance their ability to uncover patterns within matrix data and possess the potential for solving matrix-related tasks.

Although DS exhibits certain limitations and seq2seq models with DS currently face instability in terms of inference accuracy, there is a need for improvement to facilitate practical applications. For future endeavors, we intend to introduce a neural symbol approach that leverages a priori symbols within the model to represent matrix knowledge. This proposed approach will enable the utilization of the computational capabilities of seq2seq models for broader application domains.

## Data Availability

All the data utilized for analysis in this study were generated using custom code, thereby eliminating the need for any publicly available third-party datasets. The code employed to reproduce the experimental results, as well as the code used for generating the data, is accessible at https://github.com/Peng-weil/ds-for-seq2seq. The trained model data and the dataset can be accessed through the following link: https://drive.google.com/file/d/1r9OVIqI5fz7m2cI5fT9DoVTW1Oe0VZl6.
